# Development of a Fast Method Using Inductively Coupled Plasma Mass Spectrometry Coupled with High-Performance Liquid Chromatography and Exploration of the Reduction Mechanism of Cr(VI) in Foods

**DOI:** 10.3390/toxics12050325

**Published:** 2024-04-29

**Authors:** Ge Song, Honglian Tan, Chuhan Cheng, Peng Li, Xinyang Sun, Yuling Zhou, Yong Fang

**Affiliations:** 1 College of Food Science and Engineering, Nanjing University of Finance and Economics, Nanjing 210023, China; sgconfident02@163.com (G.S.); thl2120202615@163.com (H.T.); chengch3618@126.com (C.C.); xinyang.sun@nufe.edu.cn (X.S.); fangyong10@nufe.edu.cn (Y.F.); 2 Key Laboratory of Tropical Fruits and Vegetables Quality and Safety for State Market Regulation, Hainan Institute for Food Control, Haikou 570314, China

**Keywords:** Cr(VI), HPLC-ICP-MS, speciation, food components, reduction

## Abstract

Hexavalent chromium (Cr(VI)) is known as the most hazardous species of chromium. Speciation analysis of Cr in foods is of a great significance for assessing its influences on human health. In this study, a fast HPLC-ICP-MS method for the determination of Cr(VI) was developed for determining the content of Cr(VI) and also investigating its transformation in foods. The developed method employs an alkali extraction and weak anion-exchange column separation for distinguishing the Cr species, facilitating accurate Cr(VI) quantification within 1.5 min. This technique was applied to determine the Cr(VI) levels in a range of food products, including yoghurt, milk powder, rice flour, orange juice, green tea, white vinegar, and whole wheat bread. The results showed that no Cr(VI) was detected in these food products. Spiking experiments revealed that the recovery rate of Cr(VI) decreased with the increase in its contact time with food products. A further exploration of Cr(VI) in various food components such as vitamin C, tea polyphenols, whey proteins, gelatin, fructose, and cellulose indicated the conversion of Cr(VI) to organic Cr(III) over a period from 20 min to 60 h. It was found that high temperatures and acidic conditions accelerated the rate of Cr(VI) conversion to organic Cr(III) in the six food components mentioned above. This evidence suggests that natural reducing substances in foods probably prevent the occurrence of Cr(VI).

## 1. Introduction

Chromium, a heavy metal, is naturally present in rocks, soils, the atmosphere, and water, and can also be found in living organisms [1]. The most common oxidation states of Cr are trivalent (III) and hexavalent (VI) [2]. Cr(III) is an essential trace element in the human body and is indispensable for normal glucose and lipid metabolism [3]. In contrast, Cr(VI) is recognized by the World Health Organization as a class I carcinogen [4]. It rapidly enters cells in the forms of CrO_4_^2−^ and Cr_2_O_7_^2−^ through anion channels in cell membranes, precipitates certain proteins in the bloodstream, and may cause skin sensitization, carcinogenicity, or genetic defects through contact, inhalation, or ingestion. The essentiality and toxicity of Cr primarily depend on its chemical forms; Cr(VI) is 100 to 300 times more toxic than Cr(III) [5]. For the general population, dietary intake represents the primary route of exposure to Cr, with foods such as meat, dairy products, bread, and tea identified as major contributors to chromium levels in human diets. Therefore, the speciation analysis of Cr in foods is crucial for understanding and managing its impact on health.

Due to the toxicity and mobility of Cr(VI), numerous studies have focused on the quantitative assessment of Cr(VI) in foods. Mathebula et al. investigated bread and breakfast cereals, finding that 33–73% of total Cr (58.17–156.1 μg kg^−1^) in bread existed as Cr(VI) [6]. Vieira et al. investigated Cr and Cr(VI) levels in various types of beer, finding that the average Cr concentrations ranged from 1.13 μg L^−1^ to 4.32 μg L^−1^, with an average Cr(VI) level of 2.51 μg L^−1^ [7]. Mehmood et al. collected 30 fish samples from Keenjhar Lake in Pakistan and detected Cr(VI) levels of 15.25 mg kg^−1^ [8]. However, some studies contest the presence of Cr(VI) in foods. For example, Novotnik et al. employed ^50^Cr(VI) and ^53^Cr(III) stable isotopes to track species interconversions during the extraction procedures, and the data confirmed that Cr(VI) does not exist in plant-based foods and suggested similar findings in animal-derived foods [9]. Vacchina et al. found that the absence of Cr(VI) in milk was probably due to its instability and reduction by antioxidants and organic matter in the samples [10]. It can be seen that the presence of Cr(VI) in food remains a contentious issue.

Previous studies have primarily focused on detecting Cr(VI) in foods, frequently neglecting the possible changes it undergoes within the intricate environments of food matrices. The behavior of Cr in food is complex; Cr(VI) is influenced by pH and redox potential, making it inherently unstable and prone to reduction [11,12]. Furthermore, the diverse nature of food matrices allows Cr(VI) to interact with a multitude of components, leading to various transformations, a process that is further complicated by different food processing techniques [13,14]. This intricate interplay highlights the necessity for an in-depth investigation into how food components influence the detection of Cr(VI). To date, research into the transformation of Cr(VI) within food components remains limited, particularly concerning the rates at which these transformations occur.

Currently, detection methods for Cr(VI) are mainly divided into chromatographic and non-chromatographic techniques. Among the non-chromatographic techniques commonly used are spectrophotometry [15], selective extraction [16], sorption procedures [17], and electrochemical methods [18]. Chromatographic techniques primarily involve flame atomic absorption spectrometry (FAAS), inductively coupled plasma optical emission spectrometry (ICP-OES) and inductively coupled plasma mass spectrometry (ICP-MS) coupled with ion chromatography (IC) or high-performance liquid chromatography (HPLC) [19,20,21,22,23]. Although various methods exist for detecting Cr(VI), HPLC-ICP-MS is the most powerful. HPLC-ICP-MS has been used for the speciation analysis of elements, including Cr, arsenic, mercury, and selenium in water, soil, seafood, and tobacco due to its advantages of continuous operation, high selectivity, wide linear range, and low detection limit [24,25]. For example, Mihai et al. performed speciation analysis using anion-exchange HPLC-ICP-MS and detected Cr(VI) within 9 min [26]. Drinčić et al. determined Cr(VI) in river sediments using morphogenic isotope dilution ICP-MS in 9 min [27]. However, current HPLC-ICP-MS methods are generally time-consuming and lack efficiency in monitoring the changes of Cr(VI) in foods. Although there are some rapid detection methods for Cr(VI), such as electrochemical techniques, fluorescence probes, and nanosensors, they struggle to address issues related to interference from complex food matrices and precise quantification of trace levels of Cr(VI) [28,29]. To more effectively track Cr(VI) changes in foods, there is a pressing need for the development of rapid and sensitive detection methods.

The aim of this study is to develop a fast and sensitive method for analyzing trace Cr(VI) levels in a variety of food matrices, and to explore how Cr(VI) is reduced across different food components. A novel anion-exchange approach was employed to optimize and establish a fast HPLC-ICP-MS method for Cr(VI) analysis. Utilizing this analytical technique, the changes in Cr(VI) levels across a variety of foods were examined. Additionally, the influence of specific food components—vitamin C, tea polyphenols, whey proteins, gelatin, fructose, and cellulose—and food processing methods on the reduction of Cr(VI) were explored. This research contributes to the detection of Cr(VI) in food items and also provides valuable insights for scientifically assessing the risks associated with Cr(VI) in foods.

## 2. Materials and Methods

### 2.1. Chemicals and Materials

Standard solutions of Cr(III) (1000 mg L^−1^) and Cr(VI) (100 mg L^−1^) were obtained from the National Institute of Metrology (Beijing, China). Analytical-grade chemicals, including magnesium chloride hexahydrate (MgCl_2_·6H_2_O), sodium hydroxide (NaOH), dipotassium hydrogen phosphate (K_2_HPO_4_), potassium dihydrogen phosphate (KH_2_PO_4_), and ethylene diamine tetraacetic acid (EDTA), were purchased from Macklin (Shanghai, China). High-purity nitric acid (HNO_3_) and ammonia water (NH_3_H_2_O) were sourced from Merck (Darmstadt, Germany). Ultrapure water was supplied by Milli-Q (Billerica, MA, USA). Food-grade chemicals, including whey proteins, gelatin, fructose, cellulose, vitamin C, and tea polyphenols were purchased from Aladdin (Shanghai, China). Seven food samples, including both plant-based and animal-derived items such as milk powder, whole wheat bread, yoghurt, fruit juice, green tea, white vinegar, and rice flour, were purchased from a local supermarket (Nanjing, China). These items were selected to ensure comprehensive analysis.

### 2.2. Sample Extraction

The extraction of Cr(VI) from food typically occurs in alkaline media, leveraging the stability of Cr(VI) at higher pH levels [30]. The extraction of Cr(VI) was slightly modified from the US Environmental Protection Agency (EPA) method 3060A [31]. Briefly, 0.5 g of the sample was weighed into a 50 mL polyethylene centrifuge tube. Then 0.5 mL, phosphate buffer solution (0.5 M K_2_HPO_4_ + 0.5 M KH_2_PO_4_), 2.5 mL alkaline solution (0.5 M NaOH + 0.28 M Na_2_CO_3_), and 0.4 g MgCl_2_ were added. The sample, after ultrasonic shaking for 20 min in a KQ-400DE ultrasonic bath (Kunshan Ultrasonic Instrument, Kunshan, China), was centrifuged at 5120× *g* for 5 min using a H1850 centrifuge (Xiangyi, Changsha, China). The clear supernatant was then filtered through a 0.22 μm mixed cellulose filter membrane (Jinteng, Tianjin, China). The obtained filtrate was then analyzed via HPLC-ICP-MS.

### 2.3. HPLC-ICP-MS Determination

The determination of Cr(VI) was conducted using HPLC-ICP-MS, with separation achieved through an anion-exchange column (Sepax Proteomix WAX-NP5 50 mm × 4.6 mm, 5 μm) connected to an Agilent 1260 series HPLC system (Agilent Technologies, Waldbronn, Germany), which included a quaternary pump, an autosampler, and a column oven. The Cr species were quantified using an Agilent 7700 series ICP-MS (Agilent Technologies, Waldbronn, Germany). The HPLC system was directly connected to the ICP-MS nebulizer through PEEK tubing. The optimum conditions of the HPLC and ICP-MS systems are detailed in Table 1.

Identification of substances was based on retention time and the signal at *m*/*z* 52. Quantification of Cr(VI) was performed using external standard curves. Nonparametric comparison tests (one-factor ANOVA test and Tukey’s HSD test) with statistical significance set at mean ± SD, n = 3, and *p* < 0.05 were conducted with SPSS Statistics 25 software (IBM, Chicago, IL, USA). Chromatograms were plotted using Origin 2018 64-bit software (Origin Lab, Northampton, MA, USA).

### 2.4. Method Performance Evaluation

The analytical methods were evaluated according to linearity, relative standard deviation (RSD), limit of detection (LOD), and recovery experiments. The high-concentration standard solution was progressively diluted to obtain mixed standard solutions of Cr(III)-EDTA and Cr(VI) at concentrations of 50 μg/kg, 25 μg/kg, 10 μg/kg, 5 μg/kg, respectively. LOD was determined by calculating three times the standard deviation (3σ) of the signal-to-noise ratio, as outlined by Letsoalo et al. [32]. The precision of the method was assessed by calculating the RSD from triplicate runs of each sample. To further investigate the transformation dynamics of Cr(VI), recovery experiments were conducted using three methods. The first involved spiking of Cr(VI) into the alkaline extraction solutions of the food samples. The second and third involved spiking of Cr(VI) into the food samples, followed by immediate (2 min) or delayed (3 h) extraction, respectively. For these recovery experiments, additions of Cr(VI) were made at concentrations of 5 μg/kg, 25 μg/kg, and 50 μg/kg.

### 2.5. Stability Study of Cr(VI) in Food Components

Quantities of 0.01 g vitamin C, 1 g whey proteins, 1 g tea polyphenols, 1 g gelatin, 1 g fructose, and 1 g cellulose were accurately weighed and each was dissolved separately in 1 L of ultrapure water. The solutions were subjected to ultrasonic shaking for 10 min and then passed through a 0.22 μm filter membrane. The samples were spiked with 200 μg/kg Cr(VI) for vitamin C and 25 μg/kg Cr(VI) for the rest. Samples were periodically analyzed to determine the Cr(VI) content, providing insight into the stability of Cr(VI) within these food components. For this purpose, the conversion rate of Cr(VI) in each food component was calculated using Equation (1):

(1) $$E=\frac{\left(C_{0}-C_{t}\right)}{C_{0}}\times 100\%$$

where E is the conversion rate of Cr(VI) (%), C_0_ is the initial Cr(VI) concentration (μg/kg), and C_t_ is the Cr(VI) concentration at time t (μg/kg).

## 3. Results and Discussion

### 3.1. Optimization of Chromatographic Separation with HPLC-ICP-MS

Chromatographic separation plays an important role in the speciation analysis of trace elements. Presently, there are two main liquid chromatographic separation modes used for separation of Cr species: reverse ion-pair mode and anion-exchange mode. In reverse ion-pair chromatography, the nonpolar stationary phase, such as octyl or octadecyl, is commonly employed, with the mobile phase typically comprising tetrabutylammonium hydroxide (TBAH) and an organic solvent such as methanol [33]. However, the use of organic solvents is not sufficiently compatible with the requirements for sample solutions in ICP-MS. In contrast, the anion-exchange mode typically employs entirely aqueous-based solutions, which allows for a mobile phase that is more compatible with ICP-MS requirements. Therefore, the anion-exchange column was considered for the determination of Cr(VI). The Proteomix WAX-NP5 chromatographic column from Sepax-tech is a kind of weak anion-exchange column. Its stationary phase is created by chemically bonding tertiary amine groups onto the hydrophilic coating surface of non-porous polystyrene/divinylbenzene (PS/DVB) particles. The Cr ions are adsorbed onto the stationary phase’s surface within the column. The extent of their interaction with the stationary phase significantly influences the elution order of the Cr peaks.

**Figure 1:**
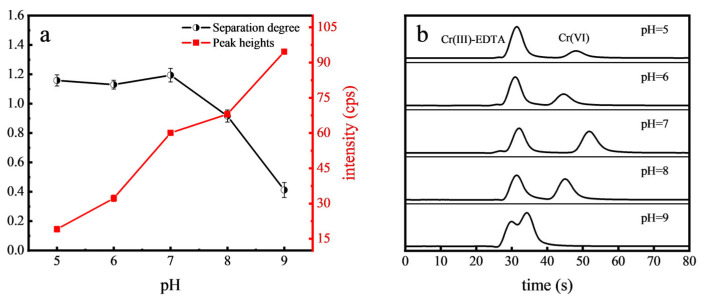
Effect of mobile phase pH on the separation degree and peak heights of Cr(VI) (**a**) and the chromatograms (**b**).

Separation degree is used to evaluate the separation effect of two peaks. When the separation degree A is less than 1.0, it is considered that the two peaks are not separated; when 1.0 < A < 1.5, the two peaks are exactly separated [34]. The pH of the mobile phase has a great influence on the existence form of Cr(VI), thereby altering its retention on the chromatographic column. This study explored the impact of pH levels ranging from 5.0 to 9.0 on Cr(VI) detection. EDTA, an effective organic ligand, was utilized with the Cr(III)-EDTA complex serving as a proxy for organic Cr for reference purposes. The peak heights of Cr(VI) and its separation degree with Cr(III)-EDTA under various pH conditions are depicted in Figure 1. It was found that the separation degree of Cr(VI) and Cr(III)-EDTA gradually decreased as the pH exceeded 7.0. At pH = 8.0, the two peaks began to overlap. This can be attributed to the decreased protonation of the stationary phase of the column and the reduced retention time under alkaline conditions [35]. Optimal separation of Cr(VI) and Cr(III)-EDTA occurred between pH 5.0 and 7.0. However, the peak heights of Cr(VI) gradually increased with the pH. Considering the balance between intensity and speed, an optimal pH of 7.0 was considered for the mobile phase in the further experiments.

**Figure 2:**
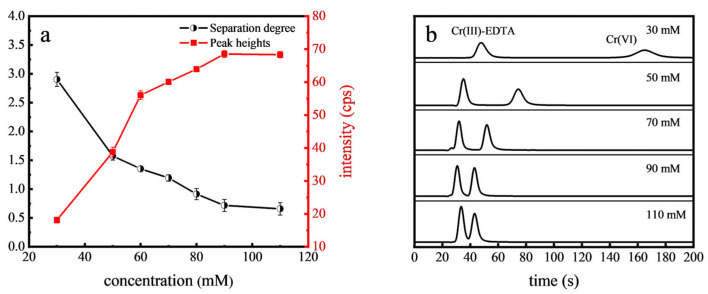
Effect of NH_4_NO_3_ concentration on the separation degree and peak heights of Cr(VI) (**a**) and the chromatograms (**b**).

The effect of mobile phase buffer on Cr(VI) separation was investigated. The buffer concentration affects the ionic strength of the mobile phase, which in turn influences the interaction between analytes and the stationary phase. NH_4_NO_3_ typically decomposes into gases such as nitrogen oxides (NO, NO_2_) and water vapor (H_2_O) under the high-temperature conditions in the ICP, minimally affecting the transportation and detection of Cr ions, making it an ideal carrier for such analysis. As depicted in Figure 2, higher concentrations of NH_4_NO_3_ increased the ionic strength, leading to shorter retention times of Cr(VI) alongside the reduced separation degree between Cr(VI) and Cr(III)-EDTA. However, the peak heights of Cr(VI) increased with the buffer concentration, stabilizing at 90 mM NH_4_NO_3_ concentration. Optimal separation and peak intensity were achieved at 70 mM NH_4_NO_3_, making it the chosen concentration for subsequent analyses.

### 3.2. Analytical Performance

Under the optimized conditions, the linear relationship and LOD of the proposed method were evaluated. A calibration curve for Cr(VI) was established within the range of 0–50 µg kg^−1^, yielding a linear equation of y = 587.32x − 24.39 with a linear correlation coefficient of 0.9996, indicating excellent linearity. The LOD, defined as three times the signal-to-noise ratio (S/N = 3), was calculated to be 0.1 µg kg^−1^. Precision, expressed through the RSD of Cr(VI) peak measurements, was assessed by repeating the analysis of a 5 µg kg^−1^ Cr(VI) standard solution five times, resulting in an RSD of 1.20%. Table 2 presents a comparison between this study and previous research. It can be seen that our method demonstrated a remarkably reduced analysis time and lower LOD. The separation and determination of Cr(VI) were achieved in just 1.5 min, significantly reducing the analysis time per sample (>70%) compared with the typical 5–11 min required for existing methods [19,35,36,37]. This efficiency gain reduces both the running costs of ICP-MS and energy usage.

### 3.3. Detection of Cr(VI) in Food Samples

**Figure 3:**
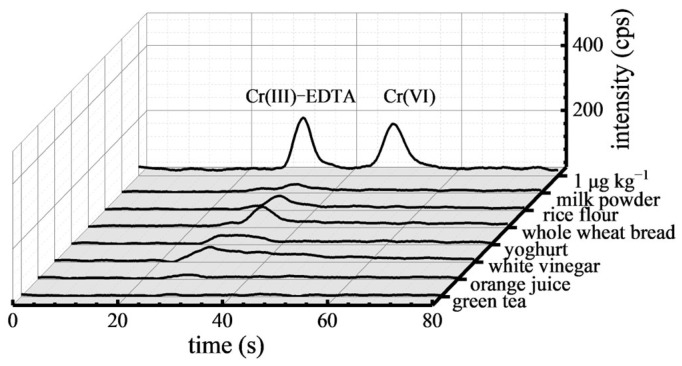
Chromatograms of Cr(VI) in the food samples.

The proposed method was used to analyze the Cr(VI) contents in various food samples such as milk powder, rice flour, whole wheat bread, yoghurt, white vinegar, orange juice, and green tea. The chromatograms of these samples are displayed in Figure 3. In comparison with 1 μg kg^−1^ Cr(VI) standard solution, it can be seen that Cr(VI) was not detected in these food samples. This suggests that Cr(VI) may not have been present in these foods.

To further investigate why Cr(VI) was not detected in any of the samples, recovery experiments were conducted using three different approaches, as described in Section 2.4. Table 3 presents the recovery rates obtained from these three spiking methods with different levels of spiking (5, 25, 50 µg kg^−1^). It was observed that when Cr(VI) was added to the alkaline extraction solutions of food samples, high recovery rates were observed. These rates ranged from 91.70% to 111.85% across different spiking levels.

Conversely, the recovery rates obtained by the spiking of Cr(VI) into the samples were relatively lower. When alkaline extraction was performed immediately after 2 min of mixing Cr(VI) with the food samples, the recovery rates ranged from 33.91% to 104.76%. In these samples, the recovery rates for milk powder, rice flour, and whole wheat bread showed little variation in comparison with the first spiking approach. However, for yogurt, white vinegar, orange juice, and green tea, the recovery rates at the low concentration of 5 µg kg^−1^ were notably reduced. For instance, in orange juice, the recovery rates at spiking concentrations of 5, 25, and 50 µg kg^−1^ were 33.91%, 90.41%, and 86.68%, respectively. When alkaline extraction was conducted 3 h after mixing Cr(VI) with the food samples, a significant decrease in the recovery rates of Cr(VI) was observed for all samples, with recovery rates ranging from 0% to 102.38%. Especially, Cr(VI) was not detected in orange juice and green tea samples. This suggests that the content of Cr(VI) in the samples decreased after mixing with food, and the decrease becme more significant over time, indicating a reduction of Cr(VI) when in contact with food components [38]. Utilizing enriched Cr isotopic spike solutions, Novotnik et al. also found that Cr(VI) was reduced in tea infusions [39].

**Figure 4:**
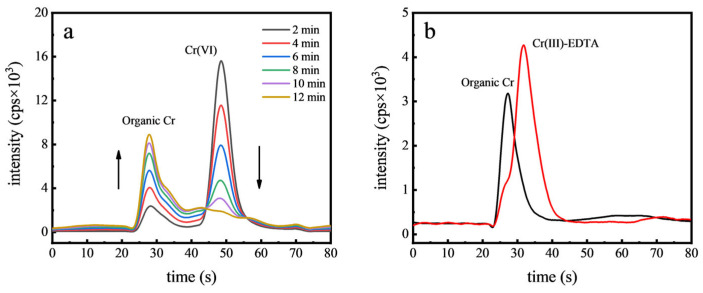
Chromatograms of Cr(VI) in orange juice with different contact time (**a**) and chromatograms after complexation with EDTA (**b**).

To test the hypothesis that Cr(VI) reduces to Cr(III) within food matrices, an experiment was conducted using orange juice. A specified concentration of Cr(VI) (50 µg kg^−1^) was introduced into the juice, and the spiked samples were subsequently analyzed using HPLC-ICP-MS. Chromatograms for samples spiked with Cr(VI) and mixed for various durations, as shown in Figure 4a, revealed that Cr(VI) converted to an unidentified Cr species within 12 min. The pH of orange juice typically ranges between 4.0 and 5.0, under which conditions the predominant form of Cr(VI) is the dichromate ion (Cr_2_O_7_^2−^). Due to the high reduction potential of Cr_2_O_7_^2−^, it is readily reduced, particularly in the presence of reducing agents such as vitamin C, which is abundant in orange juice. Vitamin C acts as a potent electron donor, facilitating the reduction of other molecules, including the conversion of Cr(VI) to Cr(III). EDTA is a potent multidentate ligand that can effectively chelate Cr(III) by forming stable complexes through its multiple electron-donating groups, thereby efficiently extracting Cr(III) from other organic compounds. To confirm that the new organic Cr peak resulted from Cr(III), 1 mM EDTA was added to chelate Cr(III). As can be seen in Figure 4b, after the EDTA addition, a distinct Cr(III)-EDTA peak emerged and the original organic Cr peak vanished. This conclusively demonstrated the rapid reduction of Cr(VI) to Cr(III) in orange juice. Similar reduction processes were also observed in the green tea samples, probably due to the presence of antioxidants like tea polyphenols, which further supports the reduction of Cr(VI) in food matrices [9].

### 3.4. Influence of Food Components and Processing on the Reduction of Cr(VI)

**Figure 5:**
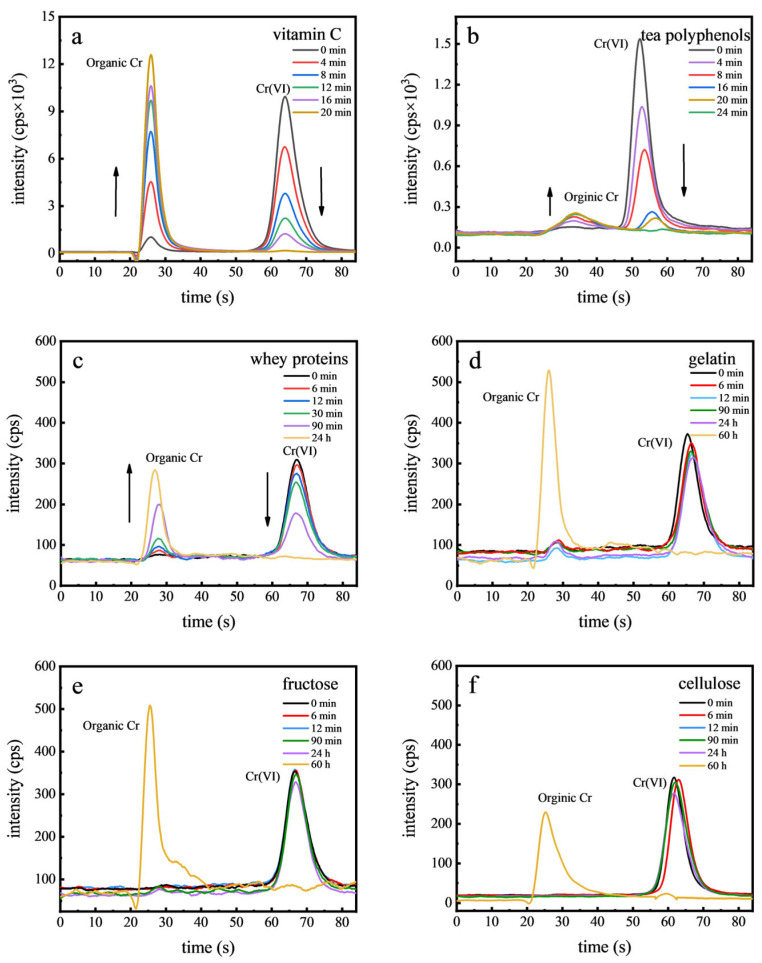
Chromatograms of Cr(VI) in food components: vitamin C (**a**), tea polyphenols (**b**), whey proteins (**c**), gelatin (**d**), fructose (**e**), and cellulose (**f**).

In the examined food samples, Cr(VI) was not detected, leading us to speculate that Cr(VI) may not typically be present in foods. To support this hypothesis, we conducted spiking experiments that demonstrated Cr(VI) is unstable and readily converts to Cr(III). This finding steered our research towards exploring the role of food components in the reduction of Cr(VI). Considering the complexity of food matrices, six representative food components were employed to study Cr(VI) stability, as shown in Figure 5. It was found that Cr(VI) rapidly converted in the presence of vitamin C and tea polyphenols, with transformation times of 20 min and 24 min, respectively. In contrast, whey proteins and gelatin exhibited slower Cr(VI) reduction rates, with Cr(VI) becoming undetectable after 24 h. For fructose and cellulose, this process took even longer, with no Cr(VI) detected after 60 h, highlighting the varying reactivity of these food components with Cr(VI). Vitamin C and tea polyphenols rapidly reduced Cr(VI) due to their abundant electron-donating functional groups, such as the enediol structure in vitamin C and hydroxyl groups in tea polyphenols, which can efficiently donate electrons and facilitate the reduction of Cr(VI) to Cr(III) [40]. Proteins such as whey proteins and gelatin, despite containing reactive amino and carboxyl groups, exhibited lower reduction efficacy due to steric hindrance and less favorable electron-donating properties. The slowest reduction rates of Cr(VI) by cellulose and fructose were primarily due to the lesser reactivity of the hydroxyl groups present in these compounds or the structural constraints that limited the interaction between Cr(VI) and the reducing sites [41]. Therefore, the presence of organics or antioxidants in food is likely to prevent the existence of Cr(VI) [42].

**Figure 6:**
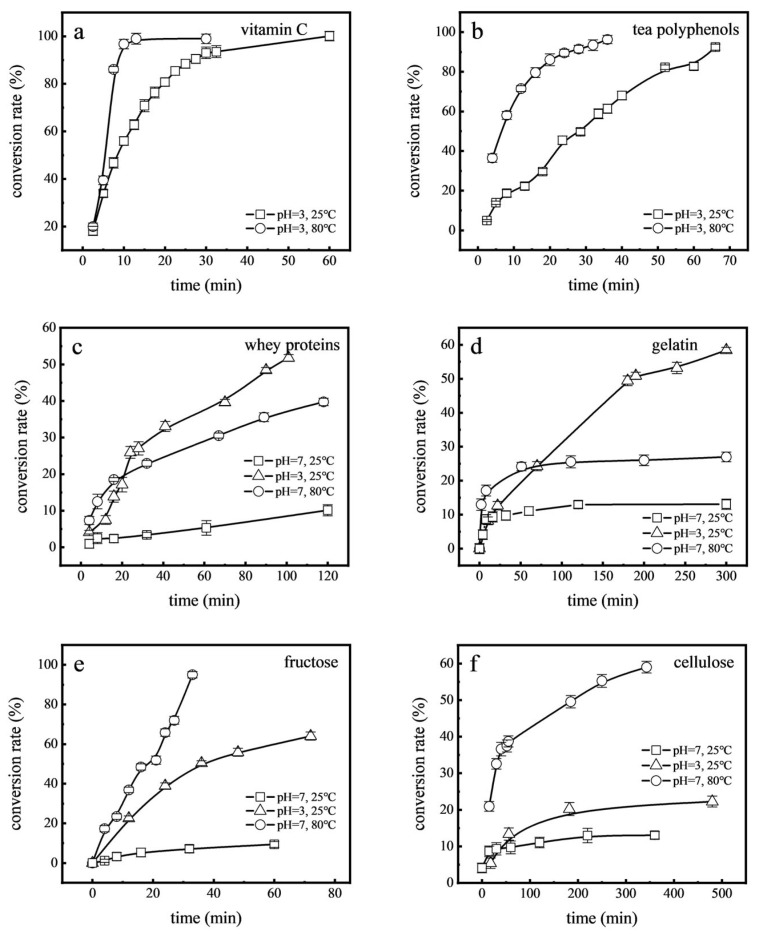
Effects of acid and temperature on the reduction of Cr(VI) in food components: vitamin C (**a**), tea polyphenols (**b**), whey proteins (**c**), gelatin (**d**), fructose (**e**), and cellulose (**f**).

Studies have shown that food processing, especially when involving contact with stainless steel, can lead to residual Cr(VI) in foods [43]. Processes such as toasting bread, cooking beef, and heat sterilization of beverages involve heating, while yoghurt and white vinegar are processed in acidic conditions. These factors raise questions about the impact of processing methods on Cr(VI) transformation. To examine this, high-temperature (80 °C) and acidic (pH 3.0) conditions were simulated to observe the reduction rates of Cr(VI) in various food components (Figure 6). It was observed that at 80 °C, compared with room temperature (25 °C), the reduction rates of Cr(VI) in vitamin C, tea polyphenols, whey proteins, fructose, gelatin, and cellulose increased by factors of 1.93, 1.04, 3.92, 10.11, 2.07, and 1.70, respectively. Higher temperature can increase the activity of atoms and molecules, increase the intensity of the reaction, and promote the reduction rate of Cr(VI) [44]. Furthermore, when compared with neutral conditions (pH 7.0), under acidic conditions (pH 3.0), the reduction rates of Cr(VI) in whey proteins, fructose, gelatin, and cellulose showed significant increases, rising 5.12, 6.81, 4.47, and 4.5-fold, respectively. This may have been due to the enhanced reactivity of reducing agents, increased availability of protons to facilitate the conversion, and the shift in Cr(VI)’s chemical forms, making Cr(VI) more susceptible to reduction [42,45]. These results illustrate the notable influence of temperature and acidity on Cr(VI) reduction rates in these food components, and that Cr(VI) detection in foods is less likely when high temperatures or acidic environments are encountered during processing.

## 4. Conclusions

In this study, a new HPLC-ICP-MS method for the fast and sensitive determination of Cr(VI) was developed and applied for the investigation of trace Cr(VI) in various foods and their relevant components. The separation of Cr species was carried out on a novel weak anion-exchange column. Under the optimal conditions of HPLC-ICP-MS, Cr(VI) exhibited a high separation efficiency, low detection limit, high precision, and, especially, a detection of Cr(VI) within 1.5 min. Cr(VI) was not detected in any of the tested food samples. The spiking recovery experiments revealed that the recovery rate of Cr(VI) decreased with the increase in its contact time with foods. This absence of Cr(VI) can be attributed to the presence of natural reducing substances in foods, which convert the Cr(VI) to Cr(III). Notably, vitamin C, tea polyphenols, and certain processing conditions, i.e., high temperature and acidity, significantly accelerated this conversion in the six food components. These findings indicate that Cr(VI) is unlikely to be present in foods, suggesting a need for cautious interpretation of Cr level data in food studies. The specific mechanisms of Cr conversion in food require further exploration.

## Figures and Tables

**Table 1:** Optimum conditions of the HPLC-ICP-MS system.

	Parameter	Settings
HPLC	Column	WAX 50 mm × 4.6 mm, 5 μm
	Mobile phase	70 mM, pH 7, NH_4_NO_3_
	Flow rate of the mobile phase	0.8 mL min^−1^
	Injection volume	5 μL
	Column temperature	Ambient
ICP-MS	Radio frequency power	1550 W
	Carrier gas flow rate	He, 1.0 L min^−1^
	Compensatory gas flow rate	1.0 L min^−1^
	Acquisition mode	Time-resolved data acquisition
	Scanning mode	Peak-hopping
	Integration mode	Peak area
	Monitored isotopes	m/z = 52(Cr)

**Table 2:** Comparison of the analytical performance of this method with previous research.

System	Column Type	Analysis Time (min)	LOD (μg kg^−1^)	References
HPLC-ICP-OES	Alltech Allsep (4.6 × 150 mm, 7 µm)	5.0	50.0	[19]
HPLC-ICP-MS	Hamilton PRP-X100 (2.1 × 150 mm, 5 μm)	11.0	30.0	[35]
HPLC-ICP-MS	Agilent ZORBAX Eclipse XDB-C8 (2.1 × 150 mm, 5 µm)	5.0	0.2	[36]
HPLC-ICP-MS	GE Healthcare Mono Q HR 5/5 (5 × 50 mm, 10 μm)	10.0	1.3	[37]
HPLC-ICP-MS	Sepax Proteomix WAX-NP5 (4.6 × 50 mm, 5 μm)	1.5	0.1	This study

**Table 3:** The experimental results for recovery rates of Cr(VI) across different food samples.

Sample	Spiked Level (μg kg^−1^)	Recovery Rates (%)		
		1: Spiking of Cr(VI) Into the Alkaline Extraction Solution	2: Spiking of Cr(VI) Into the Food Samples and Mixing for 2 min	3: Spiking of Cr(VI) Into the Food Samples and Mixing for 3 h
milk powder	5	104.67 ± 3.60 ^a^	95.57 ± 6.35 ^a^	81.88 ± 3.59 ^b^
	25	100.27 ± 1.43 ^a^	96.37 ± 3.23 ^a^	71.46 ± 3.23 ^b^
	50	102.04 ± 1.70 ^a^	100.21 ± 5.11 ^a^	61.42 ± 2.18 ^b^
rice flour	5	104.80 ± 1.54 ^a^	103.83 ± 1.41 ^a^	99.89 ± 2.05 ^a^
	25	100.91 ± 3.61 ^a^	99.47 ± 0.24 ^a^	102.38 ± 0.76 ^a^
	50	95.89 ± 1.80 ^ab^	99.33 ± 5.42 ^a^	91.96 ± 1.79 ^b^
whole wheat bread	5	105.36 ± 1.41 ^a^	103.68 ± 4.90 ^a^	85.82 ±1.41 ^b^
	25	103.97 ± 0.65 ^a^	104.76 ± 0.67 ^a^	98.76 ± 0.52 ^b^
	50	101.89 ± 5.78 ^a^	100.22 ± 6.50 ^a^	93.08 ± 0.70 ^b^
yoghurt	5	111.85 ± 2.18 ^a^	84.55 ± 4.71 ^b^	76.13 ± 5.01 ^c^
	25	107.17 ± 1.35 ^a^	92.05 ± 3.32 ^b^	78.17 ± 3.72 ^c^
	50	111.03 ± 3.01 ^a^	90.11 ± 2.43 ^b^	86.43 ± 1.42 ^c^
white vinegar	5	93.76 ± 2.18 ^a^	73.96 ± 2.13 ^b^	40.44 ± 0.37 ^c^
	25	104.30 ±1.80 ^a^	98.28 ± 1.26 ^b^	76.24 ± 0.64 ^c^
	50	103.48 ± 2.44 ^a^	93.58 ± 1.86 ^b^	76.67 ± 2.52 ^c^
orange juice	5	91.70 ± 1.48	33.91 ± 1.12	not detected
	25	105.62 ± 1.29	90.41 ± 2.14	not detected
	50	106.02 ± 2.77	86.68 ± 2.59	not detected
green tea	5	98.87 ± 1.09	79.26 ± 1.93	not detected
	25	106.92 ± 0.98	80.03 ± 0.79	not detected
	50	109.69 ± 1.72	86.88 ± 0.84	not detected

Different letters in the same row indicate a significant difference (*p* < 0.05).

## Data Availability

Data are contained within the article.
